# Distinct roles of NMB and GRP in itch transmission

**DOI:** 10.1038/s41598-017-15756-0

**Published:** 2017-11-13

**Authors:** Li Wan, Hua Jin, Xian-Yu Liu, Joseph Jeffry, Devin M. Barry, Kai-Feng Shen, Jia-Hang Peng, Xue-Ting Liu, Jin-Hua Jin, Yu Sun, Ray Kim, Qing-Tao Meng, Ping Mo, Jun Yin, Ailin Tao, Rita Bardoni, Zhou-Feng Chen

**Affiliations:** 1 Center for the Study of Itch, Washington University School of Medicine, St. Louis, MO 63110 USA; 2 Department of Anesthesiology, Washington University School of Medicine, St. Louis, MO 63110 USA; 3 Department of Psychiatry, Washington University School of Medicine, St. Louis, MO 63110 USA; 4 Department of Developmental Biology, Washington University School of Medicine, St. Louis, MO 63110 USA; 5 Department of Pain Medicine, The State Key Clinical Specialty in Pain Medicine, The Second Affiliated Hospital, Guangzhou Medical University, Guangdong, 510260 P.R. China; 6 Guangdong Provincial Key Laboratory of Allergy & Clinical Immunology, The State Key Laboratory of Respiratory Disease, The Second Affiliated Hospital, Guangzhou Medical University, Guangzhou, Guangdong, 510260 P.R. China; 7 Department of Neurosurgery, Xinqiao Hospital, Third Military Medical University, Chongqing, 400037 P.R. China; 8 Department of Biomedical, Metabolic and Neural Sciences, University of Modena and Reggio Emilia, Modena, 41125 Italy; 9 Present Address: Department of Anesthesiology, The First Hospital of Yunnan Province, Kunming, Yunnan 650031 P.R. China; 10 Present Address: Department of Anesthesiology, Plastic Surgery Hospital, Chinese Academy of Medical Sciences and Peking Union Medical College, Beijing, 100144 P.R. China; 11 Present Address: Department of Anesthesiology, Shanghai Ninth People’s Hospital, Shanghai Jiao Tong University School of Medicine, Shanghai, 200011 P.R. China; 12 Present Address: Department of Anesthesiology, Renmin Hospital of Wuhan University, Wuhan, Hubei 430060 P.R. China; 13 Present Address: Department of Anesthesiology, the Affiliated Nanhai Hospital of Southern Medical University, Foshan, Guangdong, 528000 P.R. China

## Abstract

A key question in our understanding of itch coding mechanisms is whether itch is relayed by dedicated molecular and neuronal pathways. Previous studies suggested that gastrin-releasing peptide (GRP) is an itch-specific neurotransmitter. Neuromedin B (NMB) is a mammalian member of the bombesin family of peptides closely related to GRP, but its role in itch is unclear. Here, we show that itch deficits in mice lacking NMB or GRP are non-redundant and *Nmb/Grp* double KO (DKO) mice displayed additive deficits. Furthermore, both *Nmb/Grp* and *Nmbr/Grpr* DKO mice responded normally to a wide array of noxious stimuli. Ablation of NMBR neurons partially attenuated peripherally induced itch without compromising nociceptive processing. Importantly, electrophysiological studies suggested that GRPR neurons receive glutamatergic input from NMBR neurons. Thus, we propose that NMB and GRP may transmit discrete itch information and NMBR neurons are an integral part of neural circuits for itch in the spinal cord.

## Introduction

The spinal cord dorsal horn is comprised of multiple micro neural circuits, which may function through cascades, in parallel, or in an overlapping manner. Itch and pain are transmitted from the periphery by the dorsal root ganglion (DRG) neurons to the spinal cord. Projection neurons of lamina I and V send the signals to the supraspinal sensory nuclei, and evidence indicates that itch and pain signals are processed by modality specific interneurons to shape projection output of the spinal cord. Understanding how itch and pain information is encoded and transmitted by a myriad of neuropeptides as well as by discrete neural circuits, however, poses a significant challenge^1–5^. Central to the challenge is the question of whether there are itch-specific neurotransmitters and neural circuits (pruriceptors), and if so, how they transmit different types of itch information from primary pruriceptor afferents to the brain. Among numerous neuropeptides identified in DRGs, GRP has emerged as a putative itch-specific neuropeptide^3,6–9^. The role of GRP-GRPR signaling is largely restricted to nonhistaminergic itch^6,10,11^, including opioid-induced itch^8^. Although GRPR may compensate for histaminergic itch, this mechanism can be explained by a cross-signaling model rather than the actual requirement for GRPR in histamine-induced itch^12^. At the circuit level, spinal neuronal ablation and behavioral studies suggested that spinal GRPR neurons constitute a central itch-specific circuit^7,13–16^. Rendering further support for the role of GRP-GRPR signaling in itch, we recently reported that GRP/GRPR in suprachiasmatic nucleus is also required for contagious itch behavior^17^.

Neuromedin B (NMB), another member of the mammalian bombesin peptide family, is more broadly expressed than GRP in DRGs, predominantly in Isolectin B4 Griffonia simplicifolia- (IB4)-binding neurons^12,18,19^, which was thought to be non-peptidergic neurons^20^. NMBR interneurons are mostly glutamatergic and intermixed with GRPR neurons in laminae I-II of the spinal cord^12^. Past studies have shown that intrathecal (i.t.) injection of NMB elicits dose-related scratching behavior with a rapid onset profile, indicating a direct activation of NMBR by NMB in the spinal cord^12,21–23^. GRP can bind to NMBR or NMB to GRPR with lower affinity than their respective cognate receptors^24^. Behavioral studies suggested that NMB acts exclusively through NMBR to relay itch information, whereas GRP can cross-activate NMBR as well in the spinal cord^12^. However, NMB-NMBR signaling has also been implicated in pain transmission^25^, raising an important question as to how NMB may exert its effects on both itch and pain transmission. Lack of a role of GRP in nociceptive processing has been suggested by normal pain behaviors of mice lacking *Grp* ^6^. The possibility that NMB may compensate for the loss of GRP in nociceptive processing, however, has yet to be examined.

To address the issue, we generated and analyzed the phenotype of *Nmb* KO mice, *Nmb*/*Grp* double knockout (DKO) mice and *Nmbr/Grpr* DKO mice. We also studied the role of NMBR neurons in itch and pain and their relation with GRPR neurons using behavioral and electrophysiological approaches. Our studies suggest that spinal NMB-NMBR and GRP-GRPR pathways encode discrete itch information. Moreover, our data suggest that NMBR neurons may function upstream of GRPR neurons via glutamatergic transmission.

## Results

### Distinct requirement for NMB and GRP in itch transmission

**Figure 1:**
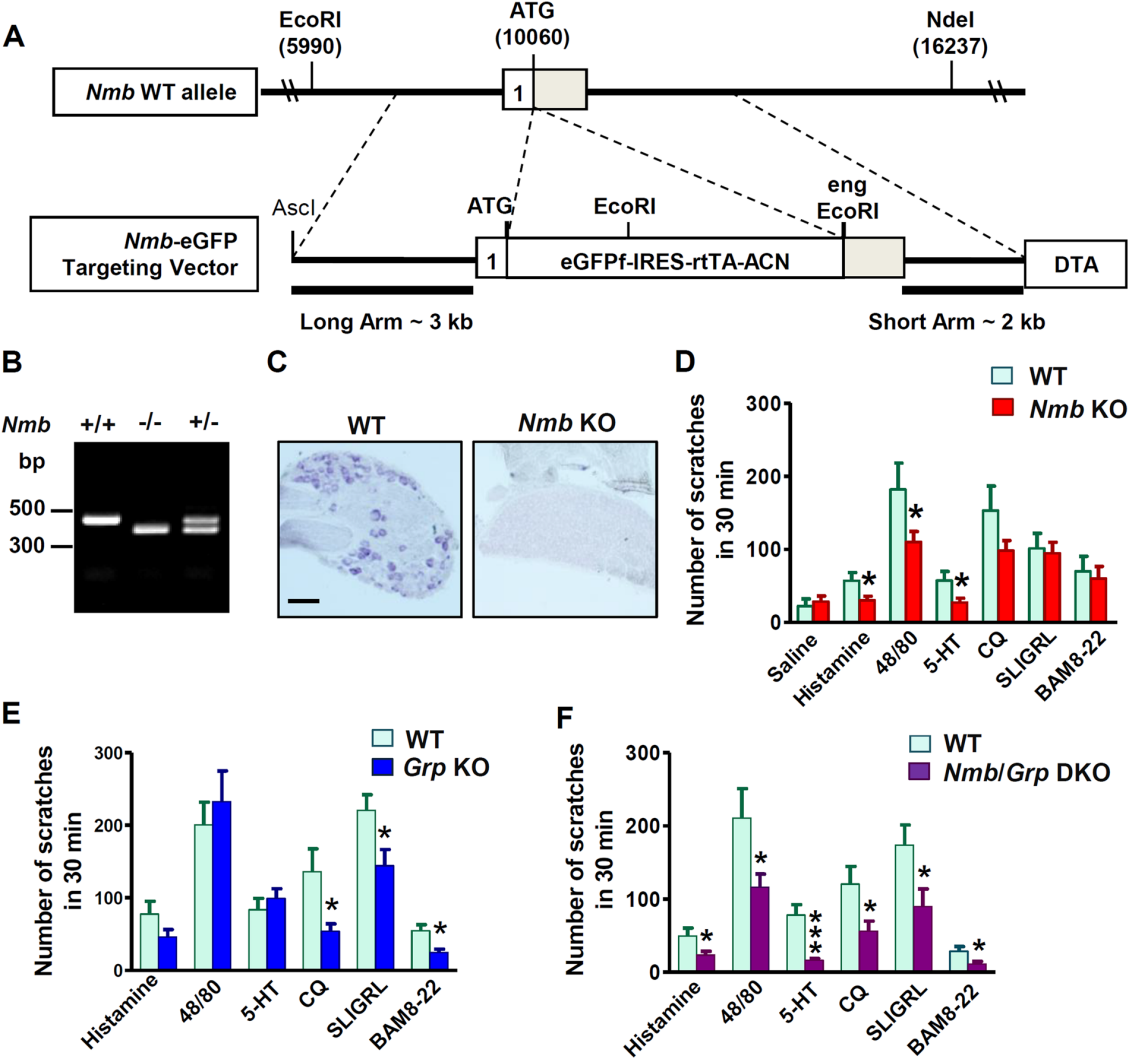
NMB and GRP are required for acute itch in a non-overlapping manner. (**A**) Schematic of gene targeting strategy of *Nmb*. Exon 1 of the *Nmb* coding region was replaced with an eGFP-IRES-rtTA-ACN targeting construct to produce a null allele. A diphtheria toxin (DTA) cassette was inserted as a negative selection marker. (**B**) Representative gel image for genotyping PCR to confirm the targeting of *Nmb* in mice. 470 bp WT and 370 bp null bands were produced, respectively. (**C**) In sit*u* hybridization showed signals of *Nmb* transcripts in WT DRG neurons that were absent in DRGs of *Nmb* KO mouse. Scale bar, 100 µm. (**D**) *Nmb* KO mice showed deficits in scratching response to histamine (200 µg, i.d.) (*P* = 0.0298), 48/80 (100 µg, i.d.) (*P* = 0.0378), and 5-HT (50 nmol, i.d.) (*P* = 0.0239), but not to CQ (200 µg, i.d.) (*P* = 0.1088), SLIGRL (100 µg, i.d.) (*P* = 0.7919) or BAM8-22 (100 µg, i.d.) (*P* = 0.7151). *n* = 6–12 per genotype. (**E**) *Grp* KO mice showed significantly attenuated scratching response to CQ (*P* = 0.0495), SLIGRL (*P* = 0.0329), and BAM8-22 (*P* = 0.0100), but not to histamine (*P* = 0.1571), 48/80 (*P* = 0.5582), or 5-HT (*P* = 0.4616). *n* = 6 per genotype. (**F**) *Nmb/Grp* DKO mice showed deficit in scratching response to histamine (*P* = 0.0256), 48/80 (*P* = 0.0373), 5-HT (*P* = 0.0002), CQ (*P* = 0.0300), SLIGRL (*P* = 0.0421), and BAM8-22 (*P* = 0.0253). *n* = 6–9 per genotype. Values are presented as mean ± SEM. *P < 0.05, **P < 0.01, ***P < 0.001, versus WT, unpaired t test.

We generated *Nmb* KO mice using a gene targeting strategy (Fig. 1A) and confirmed the absence of *Nmb* in *Nmb* KO mice by PCR (Fig. 1B) and *in situ* hybridization (ISH) (Fig. 1C). Although eGFP was fused in frame to the first coding exon of *Nmb*, eGFP fluorescence was not detectable in *Nmb* heterozygous nor KO mice. This could be attributed to disruption of cis-regulatory elements in the *Nmb* gene required for appropriate expression of eGFP. To assess the role of NMB in itch transmission, we examined the scratching behavior of *Nmb* KO mice after intradermal (i.d.) injection of several pruritogens. Surprisingly, compared with WT littermates, *Nmb* KO mice exhibited a significant attenuation in scratching responses to histamine, compound 48/80 (48/80), and 5-HT (Fig. 1D). These results are in contrast to the normal scratching behavior of *Nmbr* KO mice in response to the same pruritogens (Fig. S1)^12^. Notably, *Nmb* KO mice responded normally to three nonhistaminergic pruritogens: chloroquine (CQ), SLIGRL and BAM8-22^26^ (Fig. 1D). Mismatched phenotypes of mice lacking *Nmb* vs. *Nmbr* prompted us to examine the scratching behavior of *Grp* KO mice. Interestingly, *Grp* KO mice displayed significant deficits in the scratching responses to CQ, SLIGRL and BAM8-22, but not to histamine, 48/80 and 5-HT (Fig. 1E). Thus, in a marked contrast to *Nmb* and *Nmbr* KO mice, the phenotype of *Grp* KO mice is reminiscent of that of *Grpr* KO mice with predominant deficits in nonhistaminergic itch transmission^6,7^.

To address whether there is a functional redundancy between NMB and GRP, we next assessed the scratching phenotype of *Nmb/Grp* DKO mice. *Nmb/Grp* DKO mice showed significantly reduced scratching behaviors in response to all pruritogens tested (Fig. 1F). In contrast to *Nmbr/Grpr* DKO mice^12^, *Nmb/Grp* DKO mice overall did not display further reduction of scratching behaviors in response to acute pruritogens tested relative to *Grp or Nmb* KO mice, with the exception of 5-HT (Fig. 1F). These suggest that the roles of NMB and GRP in itch transmission are largely non-overlapping.

### Normal projection of primary afferents in *Nmb/Grp* DKO mice

Neuropeptides may be required for neurotrophic function, axonal growth and trafficking of the receptors/peptides^27^. For example, mice lacking Substance P (SP) exhibited deficits in the expression of several molecules in the dorsal horn^28^. To determine whether NMB or GRP is required for axonal growth and projection of primary afferents in the spinal cord, we examined several molecular markers using immunohistochemistry (IHC). Innervation patterns of calcitonin gene-related peptide (CGRP) positive and IB4-binding primary afferents in the superficial dorsal horn of *Nmb/Grp* DKO and WT littermate mice appear comparable (Fig. S2A), so are the patterns of SP and TRPV1 primary afferents (Fig. S2B and C). Quantitative analysis confirmed similar intensities of primary afferents for CGRP, IB4 binding, SP and TRPV1 between WT and *Nmb/Grp* DKO mice (Fig. S2D).

### NMB-NMBR and GRP-GRPR are dispensable for pain behaviors

**Figure 2:**
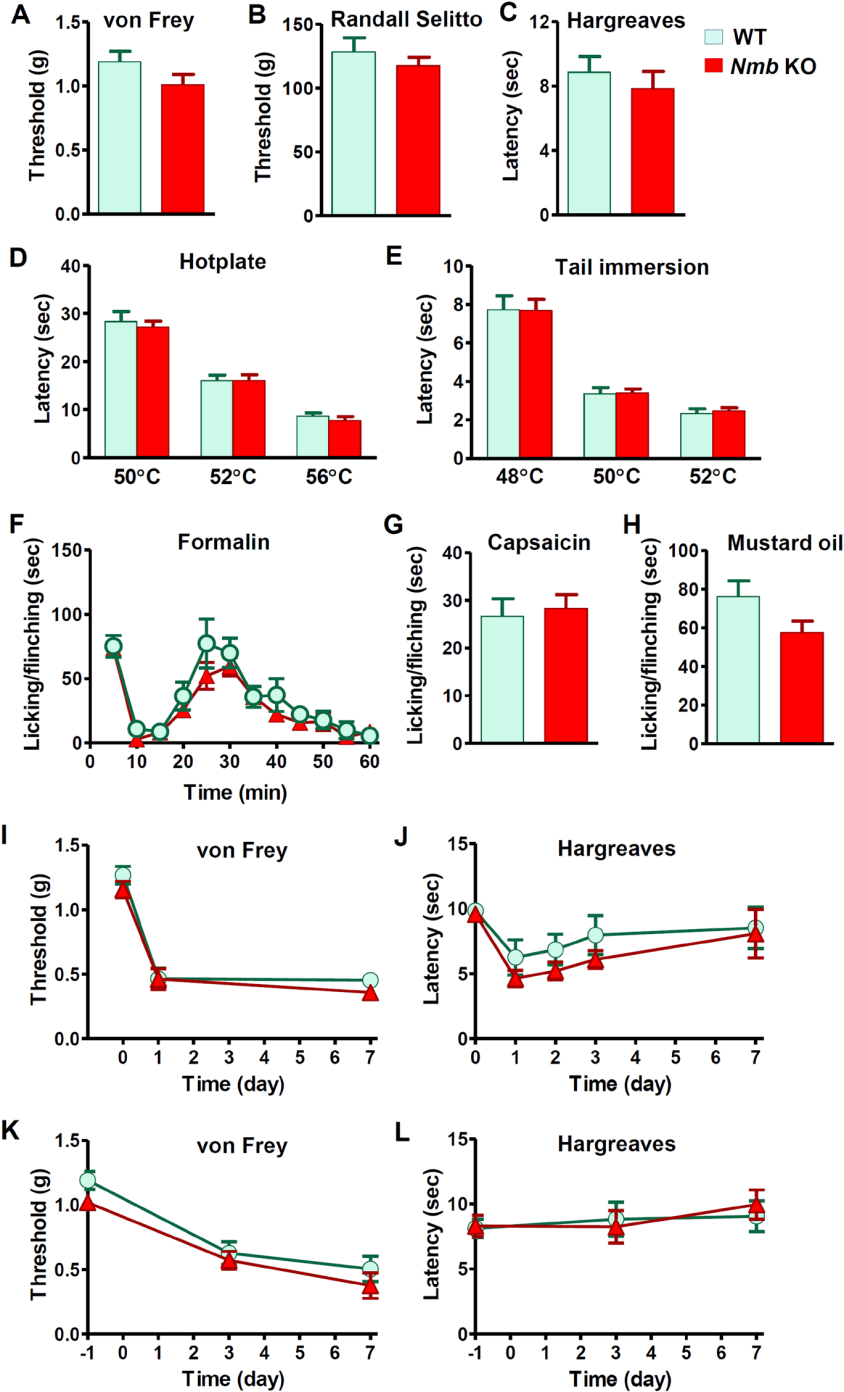
Normal pain behaviors of *Nmb* KO mice. (**A** and **B**) Mechanical pain threshold was comparable between *Nmb* KO mice and their WT littermates as tested by non-noxious von Frey assay (*P* = 0.1540, *n* = 6 per genotype) (**A**) and noxious Randall Selitto assay (*P* = 0.4072, *n* = 6–12 per genotype) (**B**). (**C**–**E**) *Nmb* KO mice showed normal response to thermal stimuli in Hargreaves (*P* = 0.4908, *n* = 6–12 per genotype) (**C**), hotplate (*P* = 0.5979, *n* = 6–9 per genotype) (**D**) and tail immersion (*P* = 0.9450, *n* = 6–9 per genotype) (**E**) tests compared with WT littermates. (**F**–**H**) Licking/flinching responses induced by 2% formalin (*P* = 0.1217, *n* = 7–10 per genotype) (**F**) capsaicin (2 µg, i.pl.) (*P* = 0.7281, *n* = 7–10 per genotype) (**G**) and mustard oil (*P* = 0.1108, *n* = 6–9 per genotype) (**H**) were not different between *Nmb* KO mice and WT littermates. (**I** and **J**) CFA induced comparable hypersensitivity to mechanical (**I**) and thermal stimuli (**J**) in both WT and *Nmb* KO mice. *n* = 6 per genotype. (**K**) After SNI *Nmb* KO mice and WT littermates developed similar extent of mechanical hypersensitivity. *n* = 6 per genotype. (**L**) SNI did not cause significant effect on thermal sensitivity of WT and *Nmb* KO mice. *n* = 6 per genotype. Values are presented as mean ± SEM. Unpaired t-test in (**A–C**, **G** and **H**), repeated measures ANOVA in (**D–F** and **I–L**).

**Figure 3:**
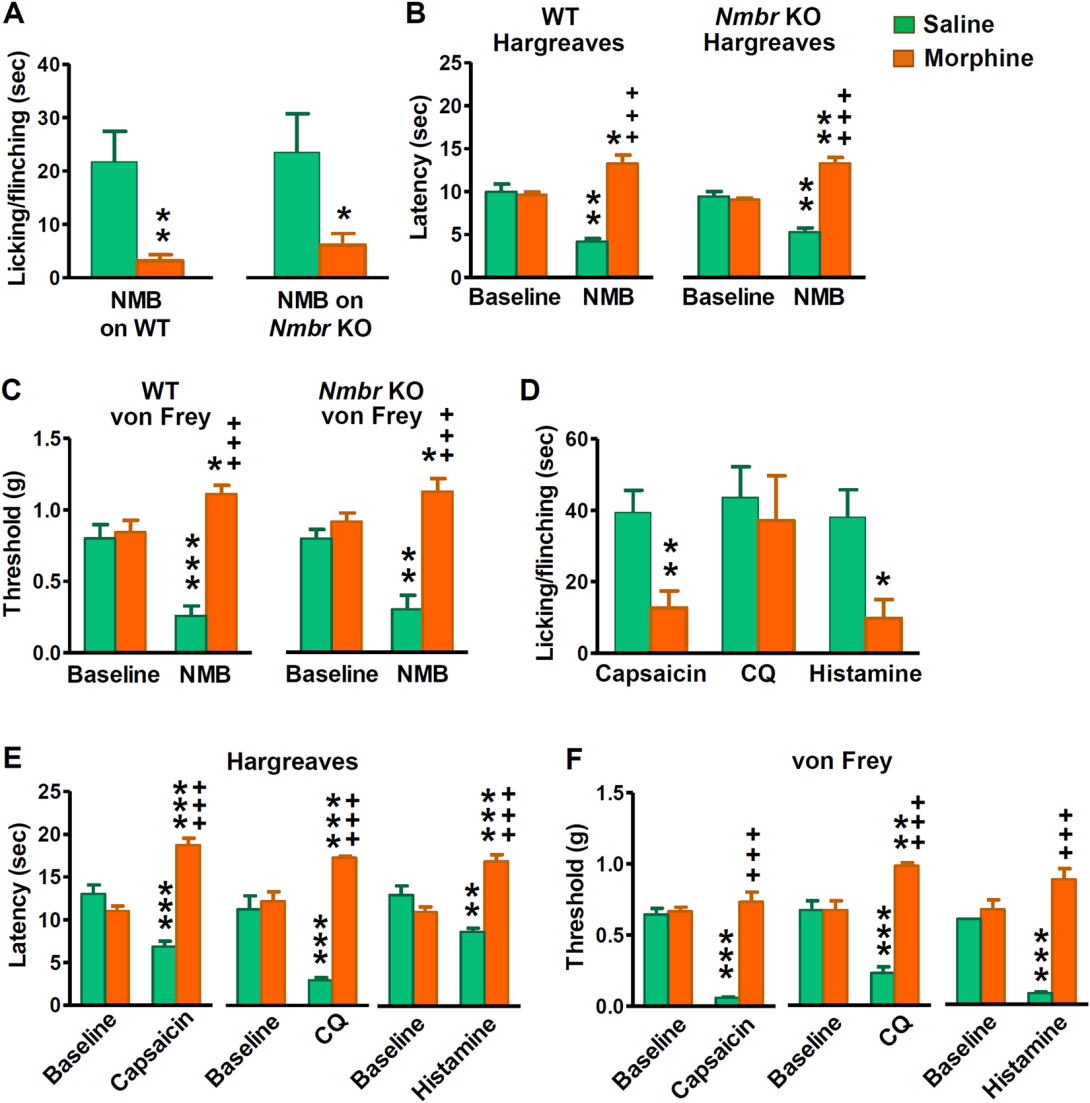
Evoked nocifensive behavior and thermal hypersensitivity after i.pl. injection of pruritogens or algogens. (**A**) Licking/flinching responses induced by NMB (45 μg, i.pl.) in WT and *Nmbr* KO mice were attenuated by pre-injection of morphine (10 mg/kg, i.p.) for 30 min. *P* = 0.0070, WT saline + NMB versus morphine + NMB, *P* = 0.0413, *Nmbr* KO saline + NMB versus morphine + NMB. *n* = 8–9 per genotype. (**B** and **C**) WT and *Nmbr* KO mice developed thermal hypersensitivity (*P* = 0.0011, WT saline + NMB versus baseline, *P* = 0.0081, *Nmbr* KO saline + NMB versus baseline)(**B**) and mechanical hypersensitivity (*P <*0.0001, WT saline + NMB versus baseline, *P* = 0.0323, *Nmbr* KO saline + NMB versus baseline) (**C**) upon i.pl. injection of NMB, which was reversed by morphine. *n* = 8 per genotype. (**D**) C57BL/B6 mice displayed licking and flinching behavior after i.pl. injection of capsaicin (2 μg), CQ (200 μg) and histamine (200 μg). Pre-injection of morphine (10 mg/kg, i.p.) for 30 min attenuated licking and flinching behavior evoked by capsaicin (*P* = 0.0049) and histamine (*P* = 0.0108), but not by CQ (*P* = 0.7632). *n* = 7 per group. (**E**) Thermal hypersensitivity induced by i.pl. injection of capsaicin (2 μg), CQ (200 μg) and histamine (200 μg) were reversed by pre-injection of morphine (10 mg/kg, i.p.) for 30 min. *n* = 6–7 per group. (**F**) i.pl. injection of capsaicin (2 μg), CQ (200 μg) and histamine (200 μg) evoked mechanical hypersensitivity that was reversed by morphine. *n* = 6 per group. Values are presented as mean ± SEM. *P < 0.05, **P < 0.01, ***P < 0.001, versus saline or baseline, unpaired *t* test.

To determine the function of NMB in nociceptive processing, we investigated a myriad of acute and inflammatory pain responses of *Nmb* KO mice. *Nmb* KO mice showed normal innocuous and noxious mechanical sensitivity as measured by graded von Frey filaments and Randall-Selitto test, respectively (Fig. 2A and B). Moreover, *Nmb* KO mice did not show deficits in thermal pain sensitivity, as measured by Hargreave paw withdrawal test (Fig. 2C), hotplate test (Fig. 2D) or tail-immersion test (Fig. 2E). Durations for licking/flinching behaviors of the inflamed paws in response to i.pl. injection of formalin (Fig. 2F), capsaicin (Fig. 3G) and mustard oil (Fig. 2H) were indistinguishable between *Nmb* KO mice and WT mice. Next we evaluated whether NMB is required for persistent pain behaviors by comparing i.pl. injection of Complete Freund’s adjuvant (CFA)-induced inflammatory pain responses between *Nmb* KO and WT mice. Both groups of mice developed a similar extent of mechanical and thermal hypersensitivity after CFA injection (Fig. 2I and J). We also compared neuropathic pain behaviors of *Nmb* KO and WT mice using a spared nerve injury (SNI) model^29^. *Nmb* KO and WT mice developed mechanical hypersensitivity to a similar extent, indicating normal neuropathic pain in mice lacking NMB (Fig. 2K). *Nmb* KO mice also showed normal thermal pain sensitivity (Fig. 2L).

Likewise, we found that *Grp* KO mice exhibited normal responses to an array of painful stimuli, including acute mechanical, thermal and noxious chemical stimuli (Fig. S3 A–J). To exclude the developmental and/or functional compensation from GRP in *Nmb* KO mice, we examined pain behaviors of *Nmb/Grp* DKO mice. *Nmb/Grp* DKO mice showed normal responses to acute mechanical and thermal stimuli as well as various algogens (Fig. S4A–H). To determine whether NMB and GRP are involved in mediating more long-lasting inflammatory pain, we studied the inflammatory pain responses of mice that received i.pl. injection of CFA. *Nmb/Grp* DKO and WT mice showed comparable thermal and mechanical hypersensitivity induced by CFA, suggesting that they are not involved in persistent inflammatory pain (Fig. S4I and J).

To evaluate whether a potential functional /signaling compensation may occur between GRPR and NMBR in nociceptive processing, as shown by normal histamine itch in *Nmbr* KO mice^12^, we examined pain behaviors of *Nmbr/Grpr* DKO Mice. Consistent with the results obtained in *Nmb*/*Grp* DKO mice, *Nmbr/Grpr* DKO mice also displayed normal innocuous and noxious mechanical pain sensitivity (Fig. S5A and B), acute thermal pain sensitivity and inflammatory nocifensive response (Fig. S5C–H). Thermal and mechanical hypersensitivity induced by i.pl. CFA was also comparable between *Nmbr/Grpr* DKO mice and WT littermates (Fig. S5I and J).

### Intraplantar injection of NMB-induced inflammation is not mediated by NMBR

On the basis of the observation that i.pl. injection of NMB caused neurogenic inflammation such as local swelling and thermal and mechanical hypersensitivity, NMB was proposed to be a novel nociceptive signaling molecule^25^. However, the specificity of exogenous NMB-induced inflammatory response was not tested. To evaluate whether NMB-induced nocifensive behaviors are specific to NMBR, we repeated i.pl. injection of NMB (45 µg) in mice. Both *Nmbr* KO and WT mice displayed licking/flinching behaviors followed by development of hypersensitivity to thermal and mechanical stimuli (Fig. 3A–C). These responses represent noxious behaviors because they were markedly attenuated by intraperitoneal injection (i.p.) of morphine (10 mg/kg, i.p.), which would reduce pain but not itch-related scratching response^30^ (Fig. 3A–C). It seems that i.pl. injection of relatively large amount of neuropeptides could invariably result in non-specific nocifensive responses. We thus conclude that observed nocifensive responses induced by i.pl NMB is not mediated by NMBR in sensory neurons.

It has been shown that injections into mouse cheek is an excellent way for distinguishing pain vs. itch by counting forelimb wiping and hind limb scratching, respectively^31^. In contrast, it was unclear whether behavioral responses evoked by i.pl. injection of neuropeptides reflect exclusively pain, or itch or both. To test this, we examined licking/flinching behaviors after i.pl. injection of algogens and pruritogens, including capsaicin, CQ and histamine and found that all chemicals invariably induced spontaneous licking/flinching behaviors (Fig. 3D). To distinguish painful response from putative pruriceptive response, we treated mice with systemic morphine. Pre-injection of morphine significantly reduced the duration of licking/flinching behaviors evoked by capsaicin and histamine, suggesting that the responses evoked by i.pl. injection of capsaicin and histamine in part reflect a nocifensive component (Fig. 3D). Interestingly, morphine failed to attenuate licking/flinching response evoked by CQ (Fig. 3D), implying that CQ-evoked response is reflective of itch sensation. Although it is difficult to separate biting from licking behaviors unequivocally, the finding that biting was not attenuated by morphine supports the contention that this behavior induced by CQ is an indication of itch rather than pain^32^. We also examined thermal and mechanical responses evoked by i.pl. injection of capsaicin, CQ and histamine. All three reagents induced thermal and mechanical hypersensitivity that was reversed by i.p. morphine (Fig. 3E and F). These results show that i.pl. injection of “classic” pruritogens activate nociceptive processing. Taken together, these data suggest that behavioral responses elicited by i.pl. injection of exogenous irritants/peptides could reflect either itch or pain or both and the effect could also be non-specific.

### Spinal NMBR^+^ neurons are important for itch but not pain behaviors

**Figure 4:**
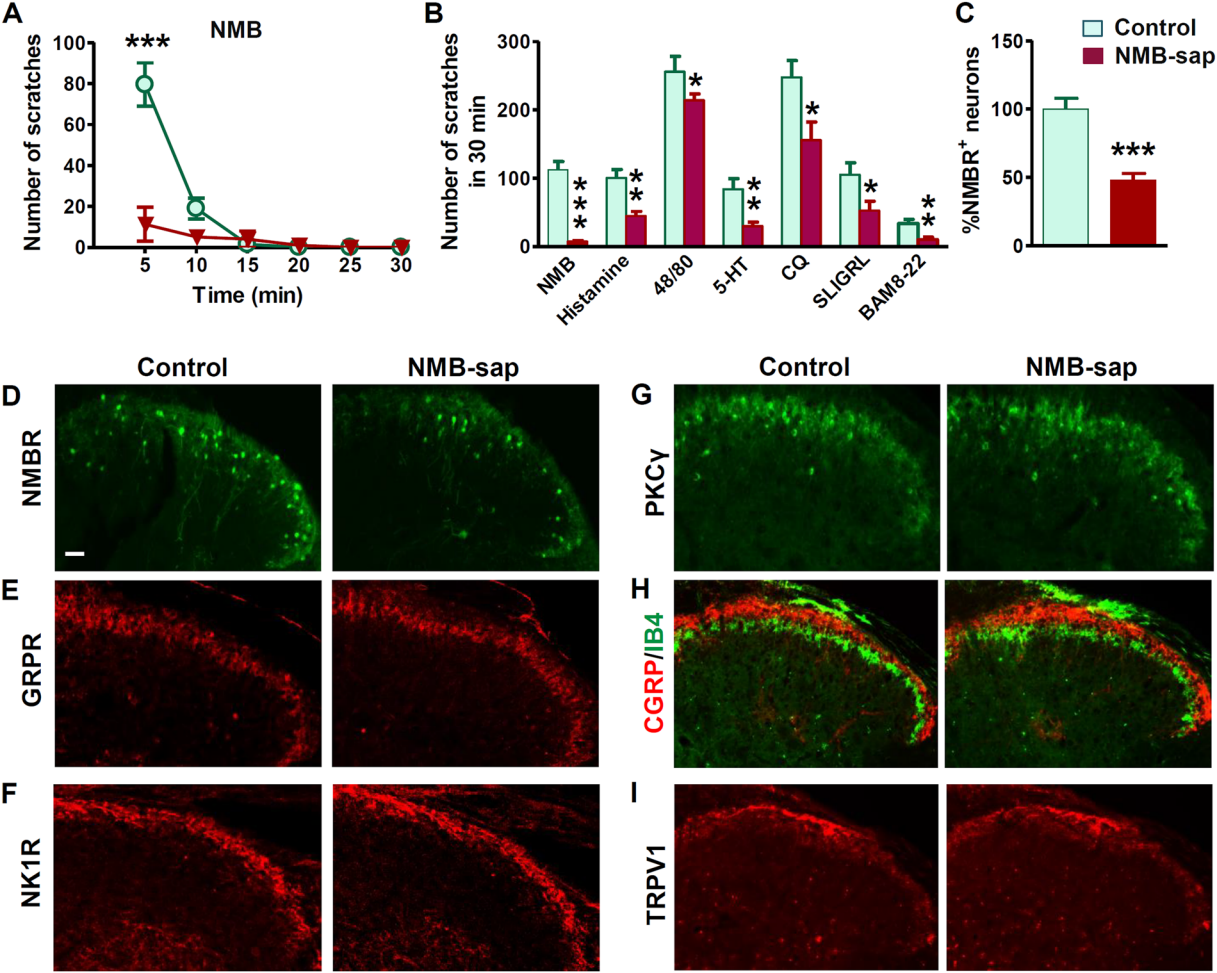
Attenuated scratching behaviours after ablation of spinal NMBR^+^ neurons. (**A**) NMB-induced scratching behavior was abolished in mice treated with NMB-sap comparing with control mice. *P* < 0.0001, repeated measures ANOVA followed by Bonferroni posttests. *n* = 6 per group. (**B**) Mice treated with NMB-sap (1–2 µg, i.t.) showed deficits in scratching response to NMB (*P* = 0.0004), histamine (*P* = 0.0240), 48/80 (*P* = 0.0487), 5-HT (*P* = 0.0301), CQ (*P* = 0.0269), SLIGRL (*P* = 0.0409), and BAM8-22 (*P* = 0.0076). *n* = 6–9 per genotype. (**C** and **D**) Quantified data (**C**) and representative images (**D**) to show decreased number of NMBR-eGFP neurons in the superficial dorsal horn of NMB-sap-treated mice. (**E**–**G**), IHC images to show GRPR neurons (**E**), NK1R neurons (**F**) and PKCγ neurons (**G**) were not affected by NMB-sap treatment. (**H** and **I**) IHC images of CGRP/IB4 (**H**) and TRPV1 (**I**) to show normal projection of primary afferents in NMB-sap-treated mice. Values are presented as mean ± SEM. *P < 0.05, **P < 0.01, ***P < 0.001, versus control. Unpaired t test in (**B** and **C**).

To examine the role of NMBR^+^ dorsal horn neurons in itch and pain transmission, we treated mice with i.t. NMB-saporin (NMB-sap, 2–3 µg) at which no side effects were observed. Taking advantage of the finding that NMB induces itch exclusively through NMBR in the spinal cord^12^, we functionally verified the loss of NMBR neurons by i.t. injection of NMB-induced scratching (NIS). NMB-sap treated mice barely showed scratching behaviors, whereas control mice exhibited robust NIS with a rapid onset of scratching responses (Fig. 4A and B), indicating that NMBR neurons were ablated in the spinal cord. NMB-sap-treated mice showed significantly attenuated scratching behaviors to i.d. injections of histamine, 48/80, 5-HT, CQ, SLIGRL and BAM8-22 (Fig. 4B). These data demonstrate an important role of NMBR^+^ neurons in itch transmission. Unexpectedly, molecular analysis using IHC revealed that the number of *Nmbr*-eGFP^+^ neurons in the superficial dorsal horn was significantly reduced, but not completely lost, in NMB-sap treated mice compared to control mice (Fig. 4C and D). By contrast, expression of GRPR, NK1R, PKCγ, CGRP/IB4 and TRPV1 was comparable to control, confirming the specificity of the ablation of NMBR neurons by NMB-sap (Fig. 4E–I). To examine whether a partial loss of *Nmb*-eGFP cells was attributable to the low dose of *Nmb*-sap we used, we repeated i.t. NMB-sap injection three times using the same dose and found that the remaining eGFP cells were not affected. Taken together with the absence of NIS in mice treated with NMB-sap, the most probably explanation is that some *Nmb*-eGFP cells do not express NMBR protein, despite the fact that spinal *Nmbr*-eGFP largely recapitulates expression of *Nmbr* mRNA^12^.

**Figure 5:**
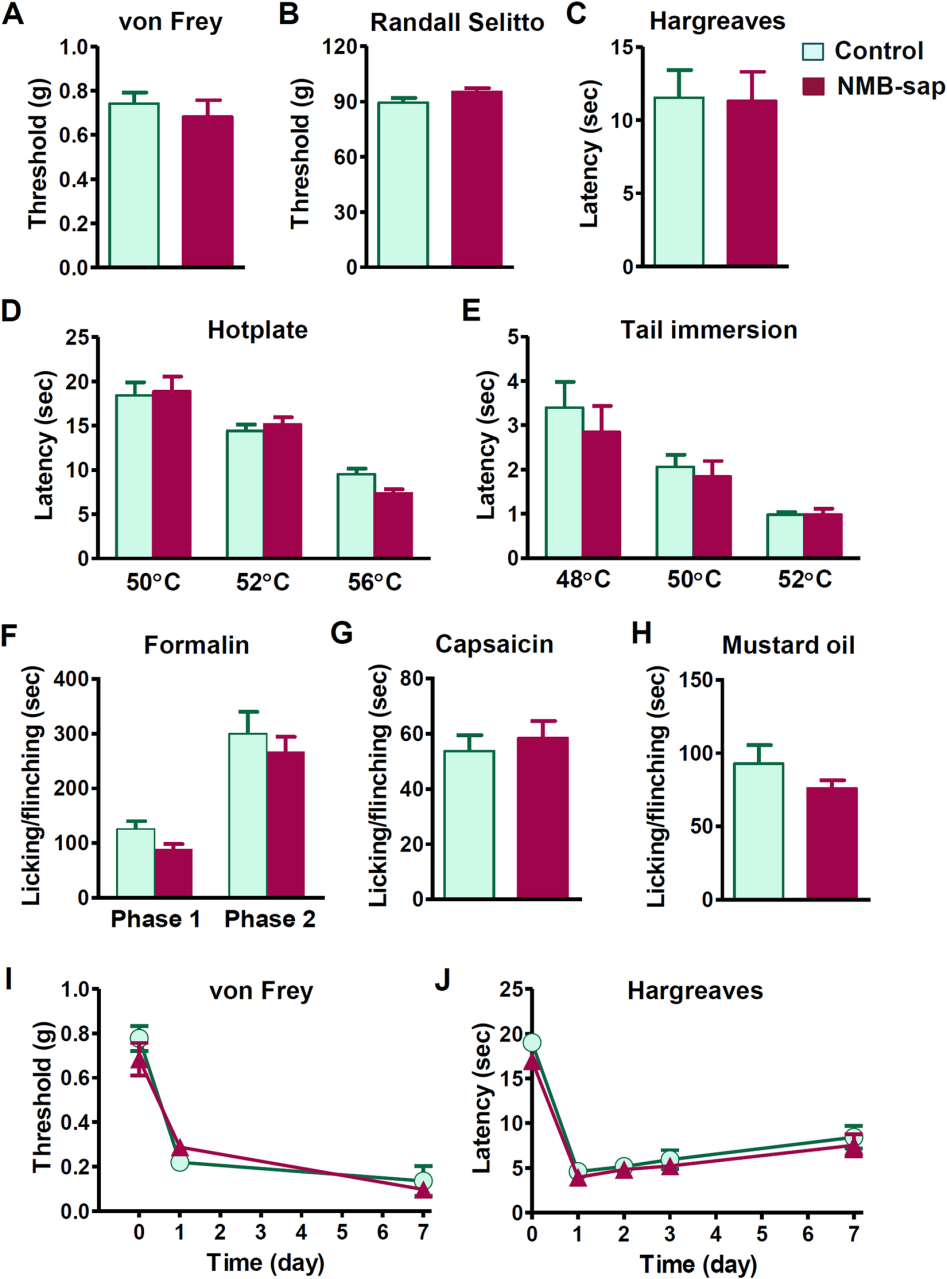
Pain behaviors of NMB-sap-treated mice. **(A** and **B**) Mechanical pain threshold was comparable between NMB-sap mice and control mice as tested by non-noxious von Frey assay (*P* = 0.5143) (**A**) and noxious Randall Selitto assay (*P* = 0.0523) (**B**). *n* = 6–10 per group. (**C**–**E**) NMB-sap mice showed normal response to thermal stimuli in Hargreaves (*P* = 0.9337) (**C**), hotplate (*P* = 0.7280) (**D**) and tail-immersion tests (*P* = 0.1223) (**E**). *n* = 6–10 per group. (**F**–**H**) Licking/flinching responses induced by formalin (*P* = 0592 for phase 1, *P* = 0.4978 for phase 2) (**F**), capsaicin (2 µg, i.pl.) (*P* = 0.7076) (**G**) and mustard oil (*P* = 0.7946) (**H**) were not different between NMB-sap mice and control mice. *n* = 7–8 per group. (**I** and **J**) CFA induced comparable hypersensitivity to mechanical (**I**) and thermal stimuli (**J**) in both control mice and NMB-sap mice. *n* = 6 per group. Values are presented as mean ± SEM. Unpaired *t* test in (**A-C** and **F-H**), two-way repeated measure ANOVA in (**D**, **E**, **I** and **J**).

In contrast to notable deficits in itch transmission, NMB-sap treated mice exhibited normal behavioral responses to acute mechanical stimuli as tested by von Frey (Fig. 5A) and Randall Selitto tests (Fig. 5B). NMB-sap treatment also failed to affect behavioral responses to thermal stimuli as tested by Hargreaves test (Fig. 5C), hotplate test (Fig. 5D) and tail immersion (Fig. 5E). We also examined inflammatory pain behaviors of NMB-sap treated mice and found normal nocifensive behaviors evoked by i.pl. injection of formalin (Fig. 5F), capsaicin (Fig. 5G) and mustard oil (Fig. 5H). I.pl. injection of CFA induced comparable mechanical and thermal hypersensitivity between NMB-sap-treated mice and control mice (Fig. 5I and J).

### GRPR neurons receive glutamatergic input from NMBR neurons

**Figure 6:**
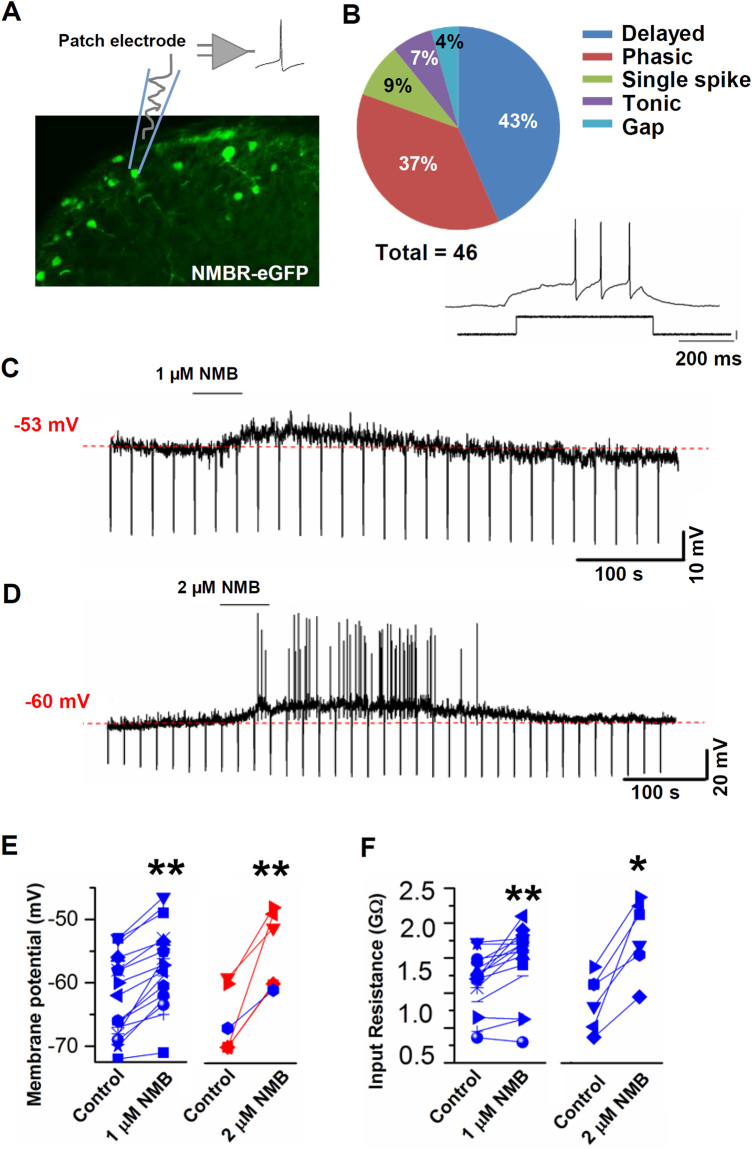
NMB depolarizes NMBR neurons and increases neuronal excitability. (**A**) Schematic diagram depicting the patch clamp approach performed on transverse sections of lumbar spinal cord of *Nmbr*-eGFP mice, where eGFP neurons are mainly located in laminae I-II (green color). (**B**) Characterization of firing patterns recorded from *Nmbr*-eGFP neurons. Positive current steps of 5–10 pA (500 ms) were applied while recording in current clamp. Delayed firing pattern was dominant (20/46). (**C** and **D**) Representative traces from the recording of NMBR neurons after NMB application. NMB was applied at the neuron resting potential (indicated in red), ranging from −59 to −70 mV. 1 µM NMB induces a subthreshold depolarization (mean depolarization: 4.9 ± 0.6 mV) (18/53), while 2 µM NMB causes action potential firing (mean depolarization: 11.1 ± 2.1 mV) (6/8). (**E**) Membrane potential changes observed in subpopulations of NMBR neurons responsive to NMB, at 2 different concentrations. In red: membrane depolarizations observed in neurons that fired AP following NMB application. (**F**) Input resistance changes induced by NMB in a subpopulation of responsive *Nmbr*-eGFP neurons. NMB caused a significant increase of input resistance at 1 µM (*P* = 0.0011) and 2 µM (*P* = 0.0127). *n* = 18 for 1 µM NMB, and *n* = 6 for 2 µM NMB. Values in (**E** and **F**) are presented as mean ± SEM. *P < 0.05, **P < 0.01, versus control, paired t test.

Next, we examined the electrophysiological properties of NMBR neurons by patch-clamp recording of *Nmbr*-eGFP neurons in spinal cord slice preparations (Fig. 6A). The delayed firing pattern was observed in most eGFP neurons, independent of their resting potential (−60 or −70 mV) (Fig. 6B). This observation is consistent with the finding that most NMBR neurons are glutamatergic, suggesting that they are primarily excitatory^12^. Application of NMB (1 µM) caused subthreshold depolarizations in most NMBR neurons (Fig. 6C and E). Increasing the NMB application to 2 µM, NMB induced depolarization and action potential (AP) firing in most NMBR neurons (Fig. 6D and E). The membrane depolarization induced by NMB was accompanied by a significant increase in input resistance at both concentrations, suggesting that the inhibition of a membrane conductance was involved (Fig. 6F). It is worth noting that irrespective of the concentration of NMB application, we found that a significant percentage of eGFP^+^ neurons did not respond to NMB, consistent with the observation that some eGFP^+^ neurons remained after NMB-sap treatment. Considering that most *Nmbr*-eGFP cells express *Nmbr* transcript^12^, these findings further support the idea that not all *Nmbr* mRNA are translated into NMBR protein, an observation reminiscent of expansion of *Grpr*-eGFP expression in chronic itch conditions^15^.

**Figure 7:**
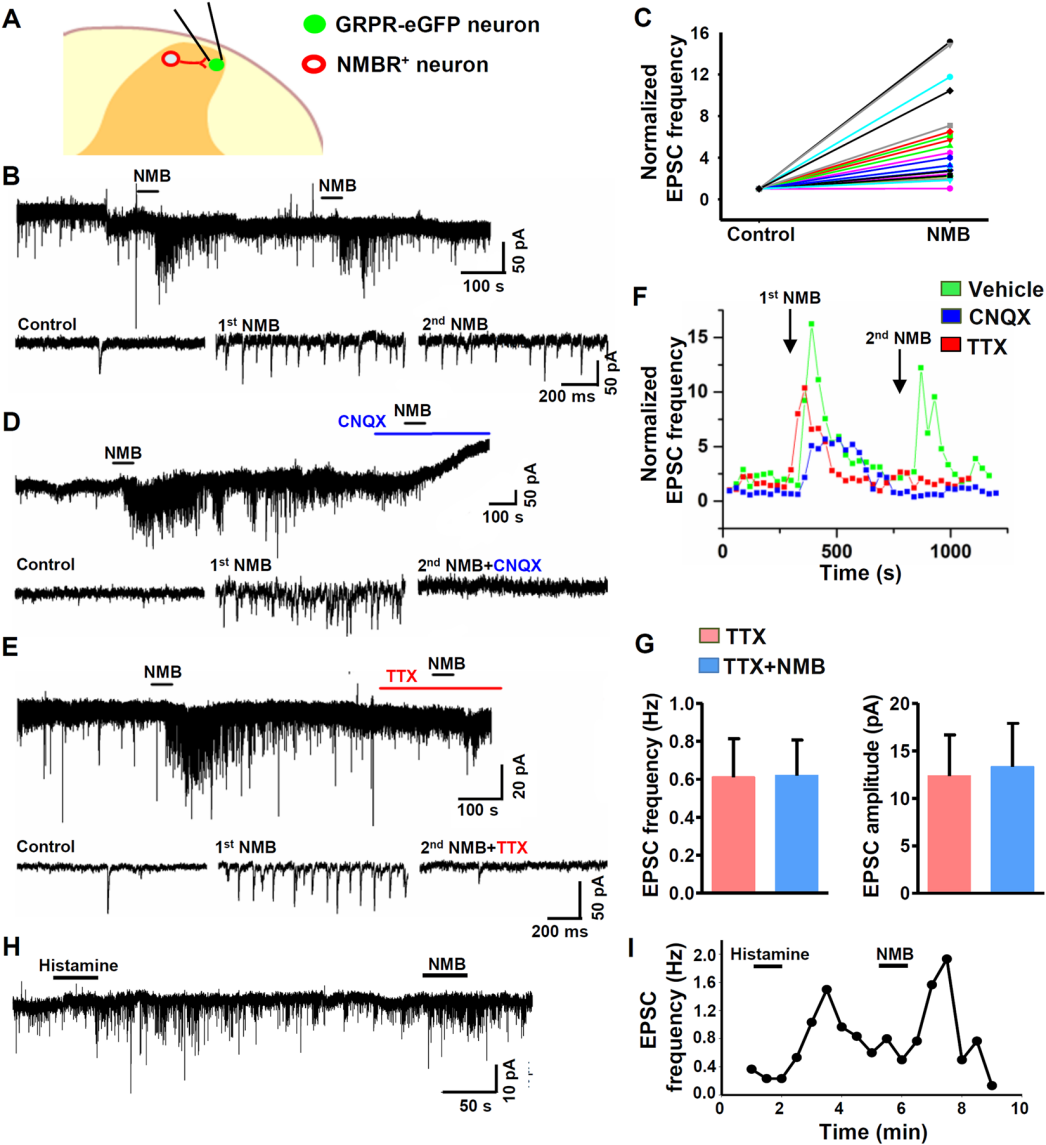
NMBR neurons provide glutamatergic input to GRPR neurons. (**A**) Schematic diagram of the dorsal horn depicting patch clamp recordings of *Grpr*-eGFP neurons that receive synaptic connections from interneurons that express NMBR. (**B**) NMB (1 µM) increases the frequency of sEPSCs recorded from a GRPR neuron (Voltage clamp recording), a 2^nd^ application of NMB increases sEPSC frequency with minimal desensitization. Lower traces depict sEPSCs on an expanded time scale for control and NMB applications. (**C**) Scatter plot of normalized sEPSC frequencies, obtained from a sample of 22 GRPR neurons responsive to NMB (22/75). NMB causes an average frequency change of 621 ± 231% (mean frequency in control: 1.5 ± 0.4 vs 6.2 ± 1.8 Hz in NMB, *n* = 22). (**D**) CNQX (50 µM) completely blocked sEPSCs recorded from a GRPR neuron responding to NMB and prevented any effect of a 2^nd^ NMB application. Lower traces depict sEPSCs on an expanded time scale for control and NMB applications. (**E**) TTX (1 µM) totally abolished the NMB-induced increase in frequency of sEPSCs recorded from a GRPR neuron. Lower traces depict EPSCs on an expanded time scale for control, 1^st^ NMB application and TTX NMB. (**F**) Representative traces depicting the effect of NMB on sEPSC frequency over time for two successive NMB applications. Green plot represents two NMB treatments. Red and blue plots represent NMB treatment followed by TTX and CNQX, respectively, obtained from 2 different neurons. (**G**) Application of NMB in the presence of TTX does not affect the frequency (*P* = 0.85) or the amplitude of miniature EPSCs (*P* = 0.12). Paired *t* test. *n* = 6. (**H** and **I**) A representative trace recorded (**H**) in voltage clamp from a GRPR neuron, showing the increase of sEPSC frequency induced by histamine (200 µM), followed by NMB (1 µM). The time course of both responses is illustrated in (**I**). Mean EPSC frequency determined for histamine-responsive neurons (11/47): 1.1 ± 0.3 Hz in control vs. 3.5 ± 0.6 Hz in histamine.

We tested whether NMBR neurons could be part of microcircuits that function upstream of GRPR neurons. Because NMB does not activate GRPR neurons directly *in vivo* ^12^, it is possible to examine the effect of NMB on GRPR neurons indirectly by recording *Grpr*-eGFP neurons^12^ (Fig. 7A). Indeed, NMB significantly increased the frequency of spontaneous EPSCs (sEPSCs), with minimal desensitization effect 10 min after the 1^st^ application (Fig. 7B, C and F). The increase in sEPSCs induced by NMB was blocked by CNQX, a non-NMDA ionotropic glutamate receptor antagonist (Fig. 7D and F). As an increased sEPSC frequency reflects enhanced glutamatergic transmission^33,34^, together these data suggest that the connectivity between NMBR neurons and GRPR neurons is glutamatergic in nature.

To determine whether the increase of sEPSC induced by NMB could result from activation of NMBR terminals of primary afferents which contact GRPR neurons^28,35,36^, we tested the effect of NMB on GRPR neurons in the presence of tetrodotoxin (TTX), a blocker of voltage-gated sodium channels. Importantly, TTX completely abolished NMB-induced increased frequency of sEPSCs in GRPR neurons (Fig. 7E and F). Under these conditions, no effect of NMB was observed on EPSC frequency or amplitude (Fig. 7G). These results suggest that NMB acts on NMBR neurons through an AP-dependent mechanism to induce the sEPSC frequency increase and in addition excludes a direct effect of NMB on AMPA receptors expressed in GRPR neurons.

Since mice lacking NMB had selective deficits in histaminergic itch, we assessed whether histamine could also increase the frequency of sEPSCs recorded from GRPR neurons, possibly by activating histamine sensitive primary afferents and increasing the excitatory drive to NMBR neurons. Indeed, histamine caused a significant increase of sEPSC frequency in a subpopulation of GRPR neurons, for the majority of which have also responded to NMB (Fig. 7H and I). The similarities between the two responses (time course and the proportion of responsive neurons) suggest that histamine and NMB could activate the same synaptic pathway, leading to an increased excitability of NMBR neurons that, in turn, release glutamate onto GRPR neurons.

## Discussion

### Spinal GRP and NMB encode itch-specific information

We found normal acute thermal, mechanical and chemical pain behaviors of mice lacking either GRP/NMB or GRPR/NMBR, suggesting that the NMB-NMBR and GRP-GRPR pathways do not compensate for each other in nociceptive processing. While NMBR and GRPR are expressed in discrete areas of the brain^37^, we did not find evidence suggesting that NMB-NMBR/GRP-GRPR signaling are major players in nociceptive processing in the nervous system. The present study represents one of the first kinds which comprehensively analyze pain behaviors of DKO mice lacking two related peptides or receptors. Together with previous findings that GRP/NMB can induce dose-dependent and itch-related scratching behavior upon i.t. injection^6,12^, the data markedly strengthen the notion that GRP and NMB are itch-specific neuropeptides. In contrast, other neuropeptides, including SP, CGRP and B-type natriuretic peptide, have been implicated in transmitting pain information from DRGs to the spinal cord, but their sites of action (interneurons vs. projection neurons; excitatory vs. inhibitory) are unclear^3,9,11,38,39^. Although a neuropeptide may be involved in both pain and itch, most likely it may exert opposing function in a state-dependent manner. An activation of the central itch circuit by an itch peptide may inhibit pain, either by interneuron-mediated cross-inhibition mechanisms^40^, by discrete subpopulations of neurons-mediated independent mechanisms^8^, or by the supraspinal descending control pathway^41^.

Considering the low dose of GRP/NMB used in dose-related response as well as scratching evoked immediately after i.t. injection^6,23^, GRP/NMB are likely to activate GRPR/NMBR directly to transmit itch information. Interestingly, it seems that the roles of NMB and GRP in itch transmission are mostly non-overlapping. This is consistent with differential expression patterns of GRP-GRPR and NMB-NMBR in both sensory neurons and spinal cord. GRPR is expressed in the superficial laminae I-II, while NMBR is mostly enriched in the lamina II inner layer^6,12,28^.

An impaired histamine-induced itch in *Nmb* KO mice confirms the role of NMB-NMBR signaling in histamine itch^12^. A cross-inhibition model was proposed to explain seemingly normal histamine-induced itch response exhibited by *Nmbr* KO mice as NMB may act as a functional antagonist for GRPR^12^. In contrast to *Nmbr* KO mice, the specific requirement for histamine-induced itch in *Nmb* KO mice was not masked. Such mismatched phenotypes indicate that lack of the ligand vs. the receptor may give rise to distinct cross-signaling dynamics. Consistent with this model, similar phenotypes in CQ- and histamine-induced itch between *Grp* and *Grpr* KO mice were observed.

**Figure 8:**
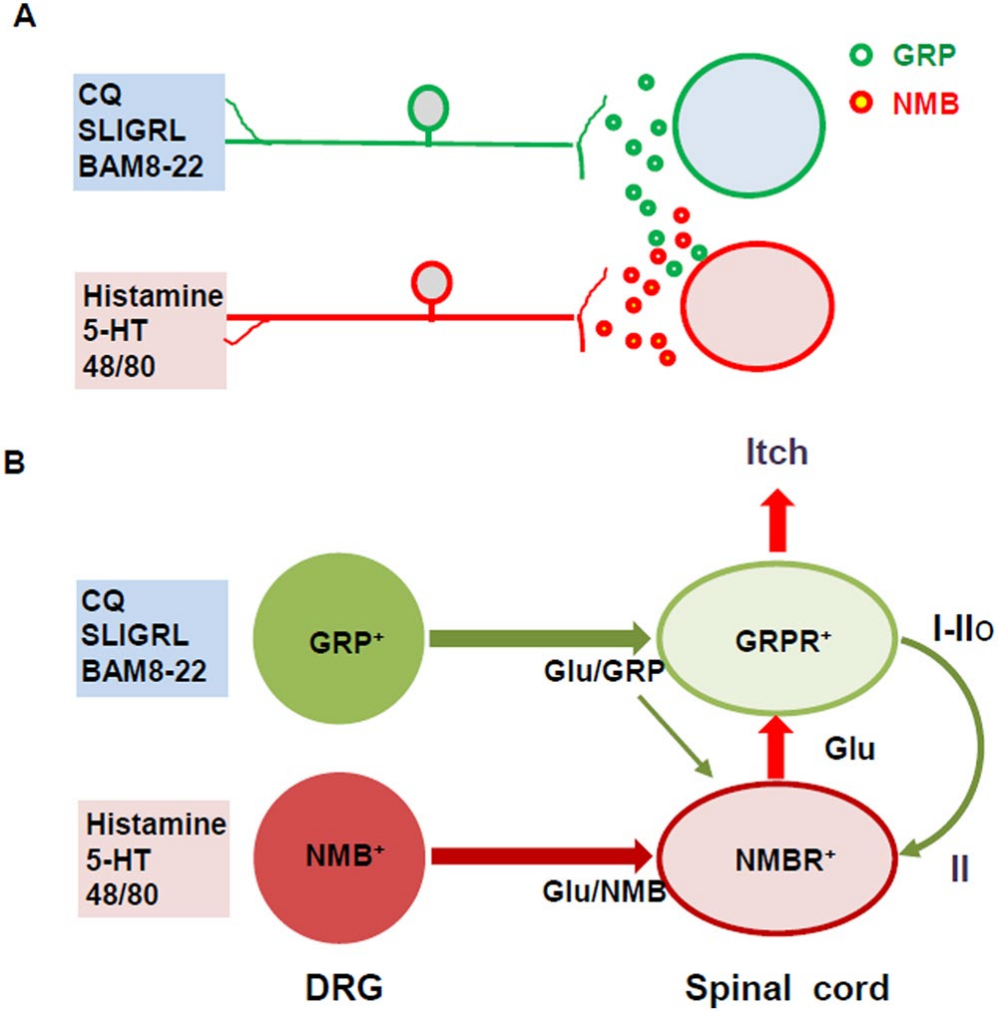
(**A**) A diagram depicting discrete pruritogenic information transmitted by GRP and NMB from sensory neurons to the dorsal horn, respectively. (**B**) A hypothetic model depicting two major itch-specific neuronal pathways that transmit itch information from DRGs to the brain via the spinal GRPR neurons. GRP fibers project to GRPR neurons that are located mainly in laminae I-II, while NMB fibers project to NMBR neurons, mostly distributed in lamina II, which relay itch information to GRPR neurons using glutamate as a transmitter. Both GRP and NMB fibers may also use glutamate as a transmitter, depending on the type of pruritogen. GRP can also activate NMBR neurons weakly. GRPR neurons and NMBR neurons form a feed forward loop in which NMBR neurons receive and amplify itch signals from GRPR neurons and send them back to GRPR neurons that function as the last output sending itch information to projection neurons. Glu: glutamate.

The observation that *Grp/Nmb* DKO mice still retained scratching behaviors in response to 48/80, CQ and SLIGRL suggests the involvement of additional neurotransmitters in itch transmission. Glutamate has been shown to be required for relaying histamine-, but not required for CQ-induced itch from DRGs to the spinal cord^42,43^. In contrast, Akiyama *et al*. showed that i.t. injection of CNQX partially attenuated CQ-induced itch^11^, implicating the role of glutamate in the process. How to reconcile these seemingly conflicting results? One explanation is that i.t. CNQX may attenuate CQ-induced itch by a blockade of glutamatergic transmission from NMBR neurons, which are activated by GRP released from primary afferents, to GRPR neurons (Fig. 8). Thus, glutamate participates in histamine- and CQ-induced itch through peripheral and central mechanisms, respectively. Consistently, we found that GRPR neurons receive monosynaptic glutamatergic EPSCs evoked by primary afferent stimulation. Thus, glutamatergic transmission is also directly involved in pruritogen-dependent itch transmission from pruriceptors in sensory neurons to GRPR neurons in the spinal cord.

### NMBR neurons function upstream of GRPR neurons in itch transmission

Our studies suggest that NMBR neurons are required for itch, but not pain transmission, and NMBR neurons function upstream of GRPR neurons. Consistent with the finding that NMBR neurons are mostly excitatory^12^, they exhibited mostly a delayed firing pattern, which is mediated by the presence of the A-type potassium current, and is mainly associated with lamina II excitatory interneurons^44–46^. NMB application induced membrane depolarization and, in some cases, action potential firing, accompanied by an increase of input resistance. The absence of an increase in sEPSC frequency and/or amplitude in GRPR neurons in the presence of TTX suggests that NMB may induce action potential-dependent glutamate release by NMBR neurons to promote synaptic excitation of GRPR neurons. Together with the behavioral observation suggesting that NMB functions exclusively on NMBR neurons *in vivo* ^12^, these data supply further evidence supporting the idea that NMB excites postsynaptic NMBR neurons directly rather than indirectly through presynaptic terminals of primary afferents. The connectivity between different subsets of excitatory interneurons in dorsal horn has been reported with uncharacterized physiological relevance^46,47^. Our finding, in contrast, is the first electrophysiological evidence illustrating the communication from NMBR neurons to GRPR neurons to transmit itch (Fig. 8).

NMBR neurons are required for a wide range of pruritogenic stimuli, including histaminergic and nonhistaminergic itch, which contrasts with a more restricted role of NMB-NMBR signaling in histamine, 48/80 and 5-HT-induced itch. The finding that 14% of NMB and GRP fibers overlap suggests that NMBR neurons can additionally receive direct inputs from GRP afferents^12^, making it possible for GRP to function as a partial agonist to transmit CQ, SLIGRL and BMA-22-induced itch via NMBR neurons^12^. On the other hand, it has been shown that GRPR neurons receive direct synaptic contacts with GRP fibers and *Mrgpra3* fibers^28,35,36^. Since NMB is expressed in both CGRP and IB4 fibers, it is conceivable that NMB and GRP afferents could simultaneously target both NMBR and GRPR neurons directly to trigger concurrent activation in response to a specific pruritogen^12^ (Fig. 8B). Together, we propose that two itch-specific neuronal pathways exist between sensory neurons and GRPR neurons as the final output in the spinal cord: an indirect NMB/GRP-NMBR-GRPR neuronal pathway and a direct GRP-GRPR neuronal pathway (Fig. 8B). A wide range of pruritogens can be detected by different subsets of sensory neurons^26,48–52^, followed by using GRPR neurons as a central hub for further process^3^. Overall, NMBR neurons play a role in itch transmission compared to GRPR neurons.

In summary, NMB and GRP appear to encode discrete itch information and NMBR neurons represent a novel itch-specific circuit which is only partially required for relaying both histaminergic and non-histaminergic itch. Together, they constitute functionally distinct but interconnected microcircuits for integrating and transmitting itch information from primary afferents to the brain. Identification of NMBR neurons as an itch circuit helps a deeper understanding of the coding logic of itch transmission in the spinal cord.

## Materials and methods

### Mice

Male mice between 7 and 12 weeks old were used for experiments. C57BL/6 J mice were purchased from the Jackson Laboratory (http://jaxmice.jax.org/strain/013636.html). C57BL/6 J mice, *Grp* KO^15^, *Nmb* KO, *Nmbr* KO mice^53^, *Grpr* KO mice^54^ and their respective WT littermates were used. Also *Nmbr* KO mice were crossed with *Grpr* KO mice to generate *Nmbr*/*Grpr* DKO mice and *Nmb* KO mice were crossed with *Grp* KO mice to generate *Nmb/Grp* DKO mice. All mice were housed under a 12 h light/dark cycle with food and water provided *ad libitum*. All experiments were performed in accordance with the guidelines of the National Institutes of Health and the International Association for the Study of Pain and were approved by the Animal Studies Committee at Washington University.

### Generation and genotyping of *Nmb* KO mice

Briefly, an *Nmb* targeting vector was generated by bacterial recombineering approach as previously described^55^. Mouse genomic 129/SvJ DNA was obtained from Sanger Institute (UK). The linearized targeting plasmid was electroporated into AB1 ES cells. Two independently targeted ES cell clones, identified by Southern blot analysis using external probes, were injected into C57BL/6 J blastocysts to generate chimeric mice. Male chimeras were mated with C57BL/6 J females to produce heterozygous mice, which were subsequently mated to produce *Nmb* KO mice and WT littermates (*Nmb* ^+/+^). The primers used for PCR genotyping were NMB-F (5′ UTR): 5′-GGACGATGCCATAAGCACGCGAGTGTGGTG-3′, GFP-R: 5′-CGGTGGTGCAGATGAACTTCAGGGTCAGCT-3′ and NMB-R (exon 1): 5′-GACTGCAGGAGCTCCGCTACCAAGAGCCTC-3′. Primer pair of NMB-F and NMB-R detects WT band of 470 bp. The band is absent in *Nmb* KO mice without exon 1. Primer pair NMB-F and GFP-R detects GFP band of 370 bp only in *Nmb* KO mice and heterozygous mice.

### Drugs and reagents

Dose of drugs and injection routes are indicated in figure legends. Histamine, 48/80, 5-HT, CQ, formalin, capsaicin, MO and Complete Freund’s Adjuvant (CFA) were purchased from Sigma (St. Louis, MO). GRP18-27 and NMB were from Bachem. SLIGRL, bovine adrenal medulla 8–22 (BAM8-22) and calcitonin gene-related peptide (CGRP_8–37_) were purchased from GenScript. Capsaicin was initially dissolved in ethanol followed by a further dilution in sterile saline. The final concentration for ethanol was 2%. Other chemicals were dissolved in sterile saline. Morphine solution (15 mg/ ml) was from WEST-WARD (Eatontown, NJ) and was diluted in sterile saline. NMB-saporin (2 µg/µl) was from Advanced Targeting Systems (San Diego, CA) and diluted in sterile saline.

### Behavioral tests

Behavioral tests were videotaped (HDR-CX190, Sony) from a side angle. The videos were played back on computer and the quantification of mice behaviors was done by persons who were blinded to the treatments and genotypes. Hind limb scratching behavior towards the injected area was observed for 30 min with 5 min intervals. One bout of scratch was defined as a lifting of the hind limb to the injection site and then replacing of the limb back to the floor or to the mouth, regardless of how many scratching strokes take place in between^6^.

### Acute scratching behavior

All behavioral tests were performed during the light cycle. Briefly, the injection area was shaved two days before experiments. Prior to the experiments, each mouse was placed in a plastic arena (10 × 11 × 15 cm) for 30 min to acclimate. Mice were briefly removed from the chamber and intradermally injected at the back of the neck.

### Mechanical sensitivity

Mechanical sensitivity was assessed using von Frey assay and Randall-Selitto assay. For von Frey assay a set of calibrated von Frey filaments (Stoelting) were used. Each filament was applied 5 consecutive times and the smallest filament that evoked reflexive flinches of the paw on 3 of the 5 trials was taken as paw withdrawal threshold. To measure tail flick threshold to noxious mechanical stimulation, a Randall-Selitto Analgesy-meter was used. Mice were held gently and the force was applied directly to the dorsal surface of the tail 2.5 cm from its end via a cone-shaped plunger. The tail flick threshold was defined as the force, in grams, at which the mouse attempts to flick its tail (cut-off force 250 g).

### Thermal sensitivity

Thermal sensitivity was determined using hotplate (50, 52, or 56 °C), Hargreaves and tail immersion assay (48, 50, or 52 °C). For the hotplate test, the latency for the mouse to lick its hindpaw or jump was recorded. For the Hargreaves test, thermal sensitivity was measured using a Hargreaves-type apparatus (IITC Inc.). The latency for the mouse to withdraw from the heat source was recorded. For the warm water tail immersion assay mice tails were dipped beneath the warm water (48, 50 or 52 °C) in a temperature-controlled water bath (IITC Inc.). The latencies to withdrawal were measured with a 20, 15 or 10-sec cutoff, respectively.

### Acute pain behavior

Different pruritogens or algogens were intraplantarly injected into the right hindpaws. The duration of licking and flinching of the injected paw was recorded for 60 min after injection for formalin test and in the first 10 min after injection for other drugs. Thermal and mechanical sensitivity of the injected paw was assessed 1 h before and 30~60 min after injection using the Hargreaves and von Frey assay, respectively. For morphine analgesia, morphine (10 mg/kg, i.p.) was given 30 min before administration of other chemicals.

### SNI

SNI was carried out according to the procedure described previously^29^. Briefly, mice were exposed to a cocktail (ketamine, 100 mg/kg and Xylazine, 15 mg/kg) to induce anesthesia. Three terminal branches of sciatic nerve were exposed, and common peroneal and the tibial nerves were cut, leaving the sural nerve intact. Muscle and skin were closed in two layers.

### IHC and ISH

Mice were anesthetized (ketamine, 100 mg/kg and Xylazine, 15 mg/kg) and perfused intracardially with PBS pH 7.4 followed by 4% paraformaldehyde in PBS. Tissues were dissected, post-fixed for 2~4 h, and cryoprotected in 20% sucrose in PBS overnight at 4 °C. Tissues were sectioned in OCT using a cryostat microtome. IHC was performed as described^56^. Briefly, free-floating frozen sections at 20 μm thickness were blocked in a 0.01 M PBS solution containing 2% donkey serum and 0.3% Triton X-100 followed by incubation with primary antibodies overnight at 4 °C, washed three times with PBS, secondary antibodies for 2 h at room temperature and washed again three times. Fluorescein isothiocyanate (FITC)-conjugated Isolectin B4 from *Griffonia simplicifolia* (IB4, 10 µg/mL; L2895, Sigma) or the following primary antibodies were used, rabbit anti-CGRPα (1:3000; AB1971, Millipore; RRID: AB_2313629), guinea pig anti-SP (1:1000; ab10353, Abcam; RRID:AB_297089), guinea pig anti- the transient receptor potential vanilloid receptor (TRPV1) (1:1000; GP14100, Neuromics; RRID:AB_1624142). The secondary antibodies were purchased from Jackson ImmunoResearch Laboratories including Cyanine 3 (Cy3)- conjugated donkey anti-rabbit or anti-guinea pig IgG (0.5 µg/ml).

ISH was performed using a digoxigenin-labeled cRNA (Roche) antisense probe for *Nmb*. Briefly, on-slide frozen DRG sections at 20 μm thickness were incubated in prehybridization solution for 3 hours at 65 °C and then incubated with *Nmb* probe (2 μg/ mL) hybridization solution overnight at 65 °C. After stringency washes, sections were incubated in PBS with 20% sheep serum and 0.1% Tween blocking solution for 3 hours and then incubated with anti-digoxigenin antibody conjugated to alkaline phosphatase (0.5 μg/mL, Roche) in blocking solution overnight at 4 °C. After washing in PBS with 0.1% Tween, sections were incubated in NBT/BCIP substrate solution at room temperature for 2~4 h for colorimetrtic detection. Reactions were stopped by washing in 0.5% paraformaldehyde in PBS. Images were taken using a Nikon Eclipse Ti-U microscope. Staining intensities were quantified by an observer blinded to the genotype using ImageJ (version 1.34e, NIH Image). At least 3 mice per group and 10 sections across each tissue were included for statistical comparisons.

### Electrophysiology

To study the synaptic input to GRPR neurons, electrophysiological experiments were performed as follows. For tissue preparation: Fresh spinal cord tissue was isolated from *Grpr*-eGFP mice under control of the *Grpr* promoter. Mice ages 17–28 days were utilized for experiments. A laminectomy was performed under an ice cold (4 °C) oxygenated (95% O_2_, 5% CO_2_) sucrose-based dissection solution (in mM, 209 Sucrose, 2 KCl, 1.25 NaH_2_PO_4_, 5 MgCl_2_, 0.5 CaCl_2_, 26 NaHCO_3_, 10 glucose) The lumbar region of the spinal cord was removed from the spinal column and embedded in agar in preparation for slicing using a vibrating tissue slicer (Leica VT 1000 S). Transverse sections of the lumbar spinal cord were obtained at a thickness of 500 µm and then stored in an incubation chamber containing oxygenated artificial cerebrospinal fluid (ACSF- containing in mM 130 NaCl, 2.5 KCl, 1.4 NaH_2_PO_4_, 1.2 MgCl_2_, 2.4 CaCl_2_, 25 NaHCO_3_, 10 glucose). For patch clamping: Spinal cord neurons were visualized under an upright microscope (Olympus BX 51) equipped with IR-DIC optics. Neurons expressing eGFP were visualized with 488 nm light (FITC filter). Spinal cord slices were mounted in a chamber (Warner RC 26 G) continuously perfused with ACSF at a rate 2 ml/min. Patch pipettes (WPI-thick wall borosilicate) were pulled (Sutter P97) to a resistance of 3–5 MΩ. Patch pipettes contained (in mM, 130 Kgluconate, 10 NaCl, 1 MgCl_2_ 0.2 EGTA, 10 HEPES, 1 MgATP, 5 NaGTP). High resistance membrane seals were made between the pipette and the membrane followed by rupture to achieve whole cell configuration. Neurons having a resting membrane potential more negative than −50 mV and an action potential amplitude of at least 80 mV were deemed healthy and viable for experiments. For current clamp experiments in NMBR neurons, firing patterns were tested with a rectangular injection of positive current in steps of 5–10 pA (500 ms). Input resistance was measured from the voltage deflection in response to injection of −20 pA (500 ms) current. NMB applications were made to neurons at resting membrane potential. To quantify excitatory synaptic responses of GRPR neurons, GRPR neurons were voltage clamped (−60 mV) in order to record synaptic currents under basal and NMB conditions. Signals for membrane potential and membrane current were controlled and amplified with a Multiclamp 700 B and Digidata 1550 A and pClamp 10.6 software. The signal for membrane current was low pass filtered at 2 kHz and digitized at 10 kHZ. Synaptic events were analyzed in Clampfit 10.6 and Minianalysis (Synaptosoft) software, membrane current traces were plotted using Origin 2015 software.

### Statistical analysis

Values are reported as the mean ± standard error of the mean (SEM). Statistical analyses were performed using Prism 6 (v6.0e, GraphPad, San Diego, CA). For comparison between two or more groups, unpaired two-tailed t-test or One-way ANOVA followed by Tukey post hoc analysis or Two-way ANOVA followed by Bonferroni posttest was used. Normality and equal variance tests were performed for all statistical analyses. Analysis of spontaneous EPSCs recorded from GRPR neurons was performed on individual neurons by using the Kolmogorov-Smirnov (K-S) test, in order to compare cumulative distributions of inter-event intervals or amplitudes. Neurons were defined as responsive when the K-S test provided a p value < 0.05. p < 0.05 was considered statistically significant.

### Data Availability

All data generated or analyzed during this study are included in this published article (and its Supplementary Information files).

## Electronic supplementary material

SUPPLEMENTARY INFO
