# Supplementary material for: Distinct roles of NMB and GRP in itch transmission
Source: Sci Rep. 2017 Nov 13;7:15466. doi: 10.1038/s41598-017-15756-0 (PMC5684337; doi:10.1038/s41598-017-15756-0)
Supplement: Supplementary file 1 — SUPPLEMENTARY INFO [file 41598_2017_15756_MOESM1_ESM.pdf]

Distinct roles of bombesin peptides in itch transmission

Li Wan[1,2,5*], Hua Jin[1,2,9*], Xian-Yu Liu[1,2,*], Joseph Jeffry[1,2,*], Devin M. Barry[1,2,*], Kai-Feng Shen[1,2,7], Jia-Hang Peng[1,2], Xue-Ting Liu[1,2,6], Jin-Hua Jin[1,2,10], Yu Sun[1,2,11], Ray Kim[1], Qing-Tao Meng[1,2,12], Ping Mo[1,2,13], Jun Yin[1,2], Ailin Tao[6], Rita Bardoni[8] and Zhou-Feng Chen[1,2,3,4].

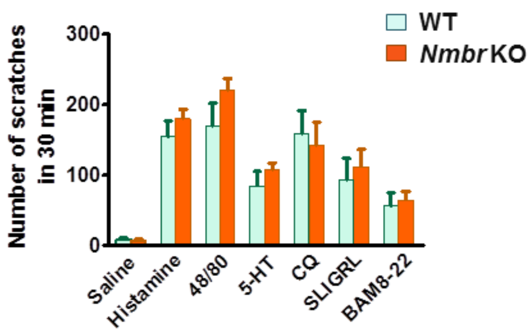

Supplementary Figure 1 *Nmbr* KO and their WT littermates showed comparable scratching responses to histamine (*P* = 0.3479), 48/80 (*P* = 0.1858), 5-HT (*P* = 0.3346), CQ (*P* = 0.7368), SLIGRL (*P* = 0.6360), and BAM8-22 (*P* = 0.7080). *n* = 6 per genotype.

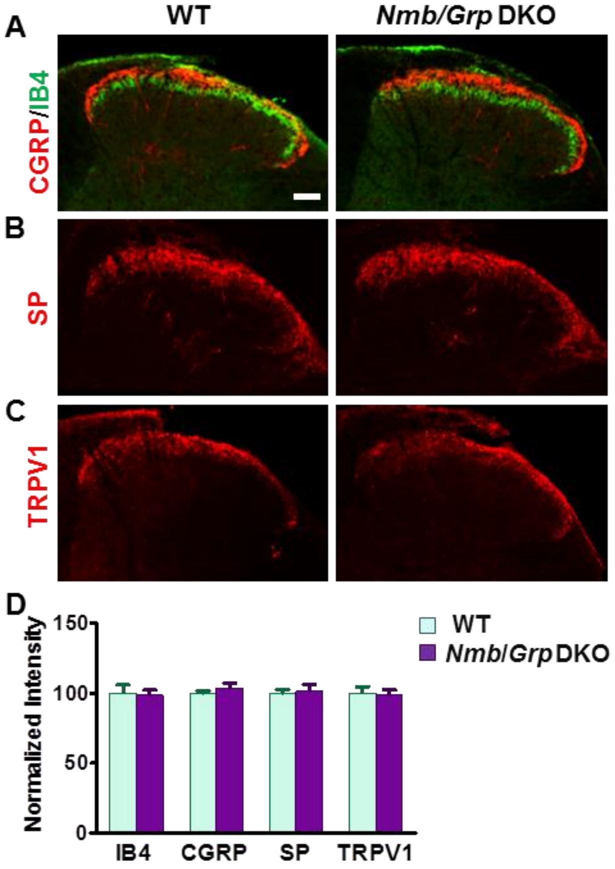

Supplementary Figure 2 Normal projection of primary afferents in the dorsal horn of *Nmb/Grp* DKO mice.

(**A**) Density and innervation of $CGRP^+$ (red) and IB4-binding fibers (green) in the superficial dorsal horn of the lumbar spinal cord is comparable between WT and *Nmb/Grp* DKO mice. (**B** and **C**) IHC of SP^+ (**B**) and $TRPV1^+$ (**C**) primary afferents in superficial dorsal horn of WT mice and *Nmb/Grp* DKO mice. (**D**) Normalized staining intensity of IB4-binding (P = 0.8477), CGRP (P = 0.4552), SP (P = 0.7632) and TRPV1 (P = 0.8790). Values are presented as mean ± SEM. n = 4 per genotype, unpaired t test. Scale bar, 100 µm.

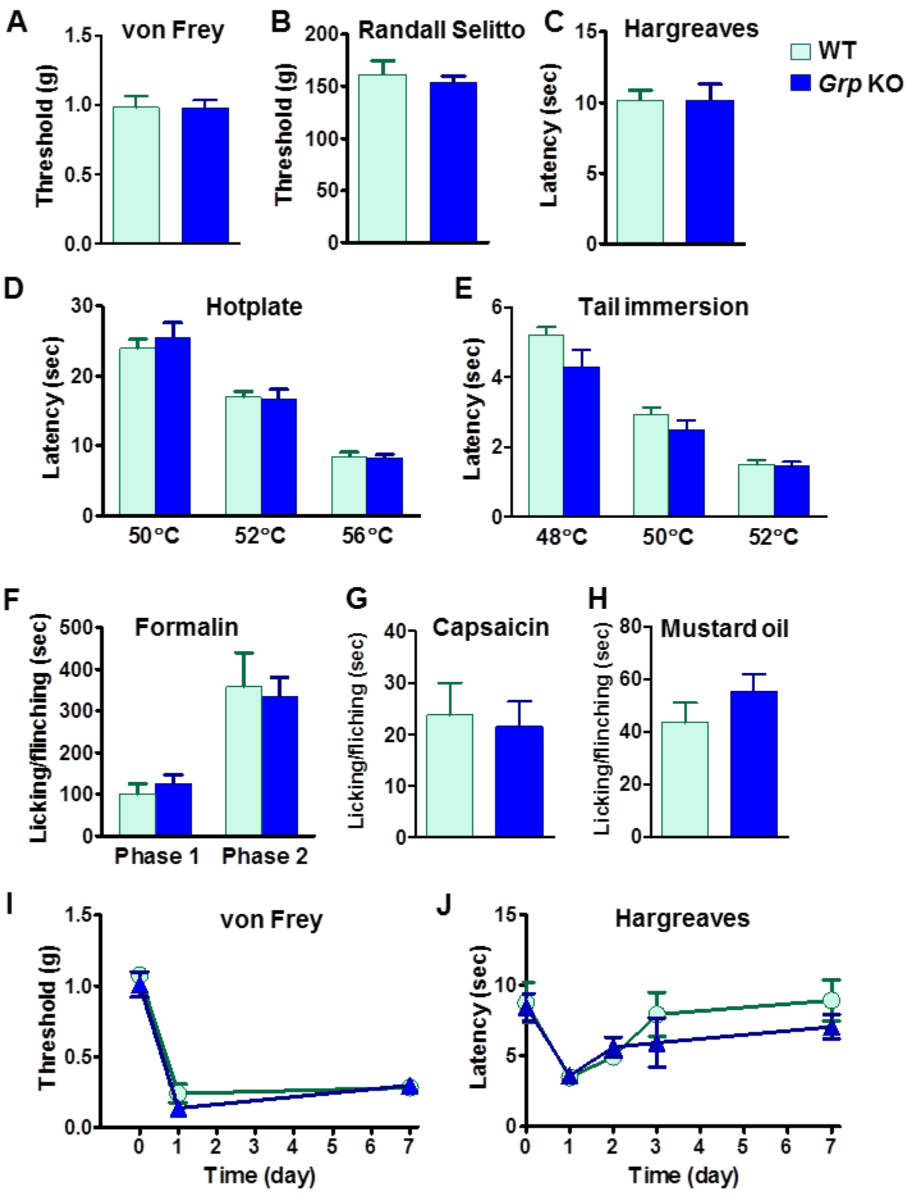

Supplementary Figure 3 Normal pain behaviors of *Grp* KO mice.

(**A** and **B**) Mechanical pain threshold was comparable between *Grp* KO mice and their WT littermates as tested by non-noxious von Frey assay (*P* = 0.9351)(**A**) and noxious Randall Selitto assay (*P* = 0.6179)(**B**). *n* = 8 per genotype. (**C-E**) *Grp* KO mice showed normal responses to thermal stimuli in Hargreaves (*P* = 0.9844)(**C**), hotplate (*P* = 0.8183)(**D**) and tail immersion (*P* = 0.1223)(**E**) tests compared with WT littermates. *n* = 8 per genotype. (**F-H**) Licking/flinching responses induced by formalin (2%, 20 µl) were comparable between WT and *Grp* KO mice (*P*

= 0.9890, *n* = 6 per genotype) (**F**), capsaicin (2 µg, 20 µl, *n* = 7 per genotype) (*P* = 0.7805)(**G**) and MO (0.75%, 20 µl, *n* = 8 per genotype) (*P* = 0.2429)(**H**) were not different between *Grp* KO and WT littermates. (**I** and **J**) *Grp* KO and WT littermates developed similar extent of mechanical (*P* = 0.3214)(**I**) and thermal hypersensitivity (*P* = 0.4659)(J) after i.pl. injection of CFA (20 µl). *n* = 7 per genotype.

Values are presented as mean ± SEM. unpaired t test in (**A-C**, **G** and **H**), two-way repeated measure ANOVA in (**D-F, I** and **J**).

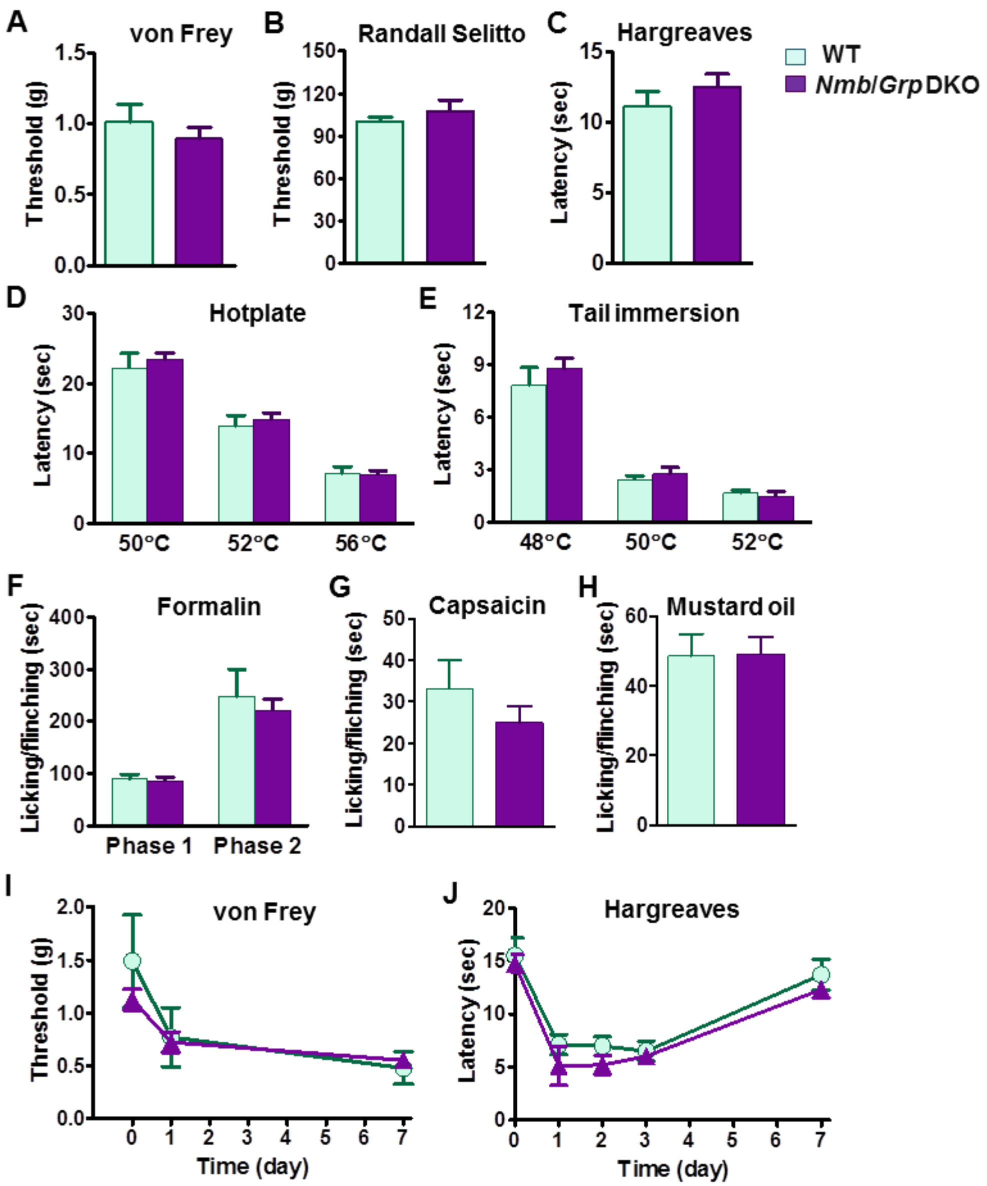

Supplementary Figure 4 Normal pain behaviors of *Nmb/Grp* DKO mice.

(**A** and **B**) Mechanical pain threshold tested by von Frey assay (P = 0.4615, n = 14-15 per genotype)(**A**) and Randall Selitto assay (P = 0.4614, n = 7-9 per genotype)(**B**) was comparable between *Nmb/Grp* DKO mice and their WT littermates. (**C**-**E**) *Nmb/Grp* DKO mice showed normal response to thermal stimuli in Hargreaves (P = 0.3589, n = 6-8 per genotype)(**C**), hotplate (P = 0.6178, n = 7-9 per genotype)(**D**) and tail immersion tests (P = 0.5485, n = 7-9 per genotype)(**E**). (**F**-**H**) Licking/flinching responses induced by formalin (2%, 20 μl) (P = 0.6178,

n = 6-7 per genotype) (**F**), capsaicin (2 µg, 20 µl) (*P* = 0.3074, *n* = 7-9 per genotype)(**G**) and MO (0.75%, 20 µl)(*P* = 0.9402, *n* = 7 per genotype)(**H**) were not different between *Nmb/Grp* DKO mice and WT littermates. (**I** and **J**) *Nmb/Grp* DKO mice and WT littermates developed comparable hypersensitivity to mechanical stimuli (*P* = 0.4348, *n* = 6 per genotype)(**I**) and thermal stimuli (*P* = 0.1406, *n* = 6 per genotype)(**J**) after i.pl. injection of CFA (20 µl).

Values are presented as mean ± SEM. unpaired *t* test in (**A-C**, **G** and **H**), two-way repeated measure ANOVA in (**D-F**, **I** and **J**).

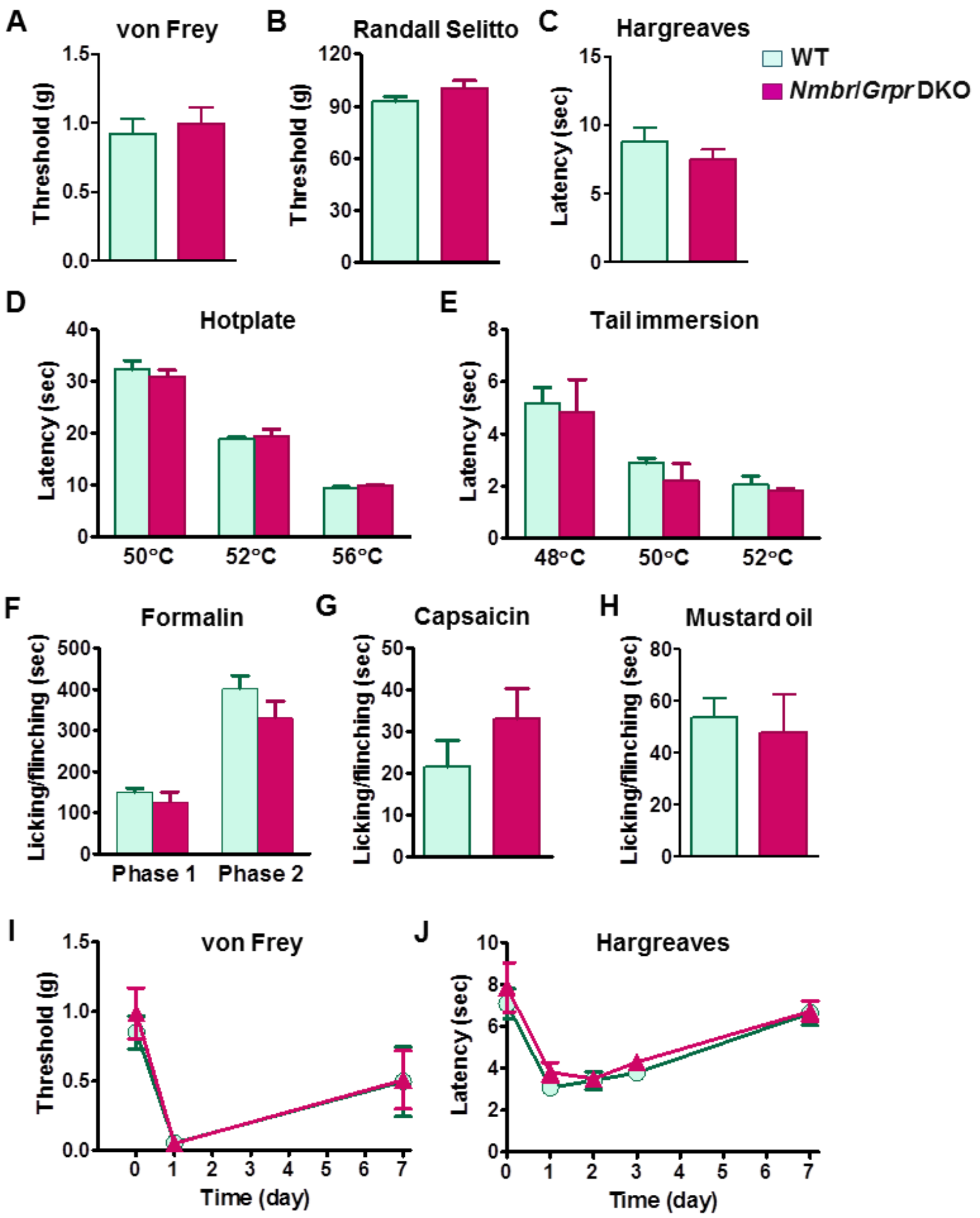

Supplementary Figure 5 Normal pain behavior of *Nmbr/Grpr* DKO mice.

(**A** and **B**) Mechanical pain elicited by non-noxious von Frey assay (*P* = 0.6511, *n* = 6 per genotype)(**A**) and noxious Randall Selitto (*P* = 0.2264, *n* = 6 per genotype)(**B**) were comparable between *Nmbr/Grpr* DKO mice and their WT littermates. (**C-E**) *Nmbr/Grpr* DKO mice showed normal response to thermal stimuli in Hargreaves (*P* = 0.3075, *n* = 12-14 per genotype)(**C**), hotplate (*P* = 0.4066, *n* = 6 per genotype)(**D**) and tail immersion tests (*P* = 0.5562, *n* = 6 per

genotype)(**E**). (**F-H**) Licking/flinching responses induced by formalin (*P* = 0.1229, *n* = 6 per genotype)(**F**), capsaicin (*P* = 0.27552, *n* = 6-7 per genotype)(**G**) and MO (*P* = 0.7216, *n* = 6 per genotype)(**H**) were not different between *Nmbr/Grpr* DKO mice and WT littermates. (**I** and **J**) *Nmbr/Grpr* DKO mice and WT littermates developed comparable hypersensitivity to mechanical stimuli (*P* = 0.7414, *n* = 6-7 per genotype)(**I**) and thermal stimuli (*P* = 0.1560, *n* = 6 - 7 per genotype)(**J**) after i.pl. injection of CFA (20 μl).

Values are presented as mean ± SEM. Unpaired *t* test in A, C, G and H, two-way repeated measure ANOVA in (**D-F, I** and **J**).
